# 100 m climate and heat stress data up to 2100 for 142 cities around the globe

**DOI:** 10.1016/j.dib.2026.112497

**Published:** 2026-01-21

**Authors:** Niels Souverijns, Dirk Lauwaet, Quentin Lejeune, Chahan M. Kropf, Kam Lam Yeung, Shruti Nath, Carl F. Schleussner

**Affiliations:** a Environmental Intelligence Unit, Flemish Institute of Technological Research (VITO), Mol, Belgium; b Vrije Universiteit Brussels (VUB), Brussels, Belgium; c Institute for Environmental Decisions, ETH Zurich, Zurich, Switzerland; d Federal Office of Meteorology and Climatology MeteoSwiss, Zurich, Switzerland; e Department of Physics, University of Oxford, Oxford, United Kingdom; f Integrated Climate Impacts Research Group, International Institute for Applied Systems Analysis (IIASA), Laxenburg, Austria

**Keywords:** Urban climate, Urban heat, Projections, High-resolution

## Abstract

Cities worldwide are increasingly facing the challenges of heat stress, a problem expected to worsen with ongoing climate change. The lack of detailed, city-specific data hinders effective response measures and limits the adaptive capacity of urban populations. In this data descriptor, we introduce a comprehensive database providing climate and heat stress information for 142 cities globally, covering the present and extending projections up to 2100 across three distinct climate scenarios, including two overshoot scenarios. This dataset includes 34 heat stress indicators at a spatial resolution of 100 meters, offering a unique database to identify vulnerable areas and deepen the understanding of urban heat risks. The data is presented through an accessible, user-friendly dashboard, enabling policymakers, researchers, and city planners, as well as non-experts, to easily visualise and interpret the findings, supporting more informed decision-making and urban adaptation strategies.

Specifications Table

**Table:** 

Subject	Earth & Environmental Sciences
Specific subject area	Microscale climate information for cities worldwide
Type of data	Spatially explicit 100 m climate information at decadal timesteps for the period 2010–2100 in NetCDF & Geotiff format
Data collection	The UrbClim urban boundary layer model is used to dynamically downscale large-scale climate information to the extent of individual cities and their rural surroundings at very high resolution (100 m). This hourly information is translated to decadal heat stress indicators for the period 2010–2100. Impact indicators are calculated using the CLIMADA model.
Data source location	142 cities around the world
Data accessibility	Repository name: Zenodo Data identification number: https://doi.org/10.5281/zenodo.13361538 Direct URL to data: https://zenodo.org/records/13361538
Related research article	None

## 1 Value of the Data

• The dataset presented in this paper provides a first-of-its kind archive of 142 cities covering all continents (excluding Antarctica) with detailed climate, heat stress and impact information for both present and future time scales (until 2100) at 100 m spatial resolution.
• The 100 m spatial resolution allows identification of the most vulnerable areas within the city and the long-term availability of the data permits calculation of heat stress impacts towards the end of the century under different emission pathways, including overshoot scenarios and their uncertainty. This provides invaluable support for urban planning, enhancing public health responses, emergency response planning and climate impact and adaptation strategies.
• The data is presented in an easy-to-access dashboard (https://climate-risk-dashboard.iiasa.ac.at/impacts/explore), allowing not only researchers, but also non-experts and policy makers to easily access, visualise and interpret the heat stress data. Lastly, a toolbox is presented to obtain similar data for cities that are currently not represented in this dataset.
• The model tools (UrbClim & CLIMADA) are validated both on temperature and humidity, important components for calculating heat stress.

## 2 Background

Heat stress is a hazard with an increasing impact, responsible for approximately 500.000 excess deaths per year worldwide [1]. In urban environments, temperatures are generally higher compared to rural environments caused by the lower amount of vegetation and abundance of sealed surfaces. Towards the future, one expects an increase in the number of heatwaves in cities [2] and their inhabitants are prospected to experience twice as much heat stress compared to rural populations [3]. Taking into account that 68 % of the global population is projected to live in urban areas by 2050 [4], heat stress in cities is a key priority to consider by policy makers, city planners and authorities. Despite the acknowledgement of the increased vulnerability of city populations to heat stress [[5], [6], [7]], current globally available datasets lack the spatial and temporal resolution to represent this additional heat burden [8,9]. The dataset presented here provides a first-of-its kind archive of 142 cities covering all continents (excluding Antarctica) with detailed climate, heat stress and impact information for both present and future time scales (until 2100) at 100 m spatial resolution, building on the work that was executed over Europe by Lauwaet et al. (2024) [10].

## 3 Data Description

**Fig. 1:**
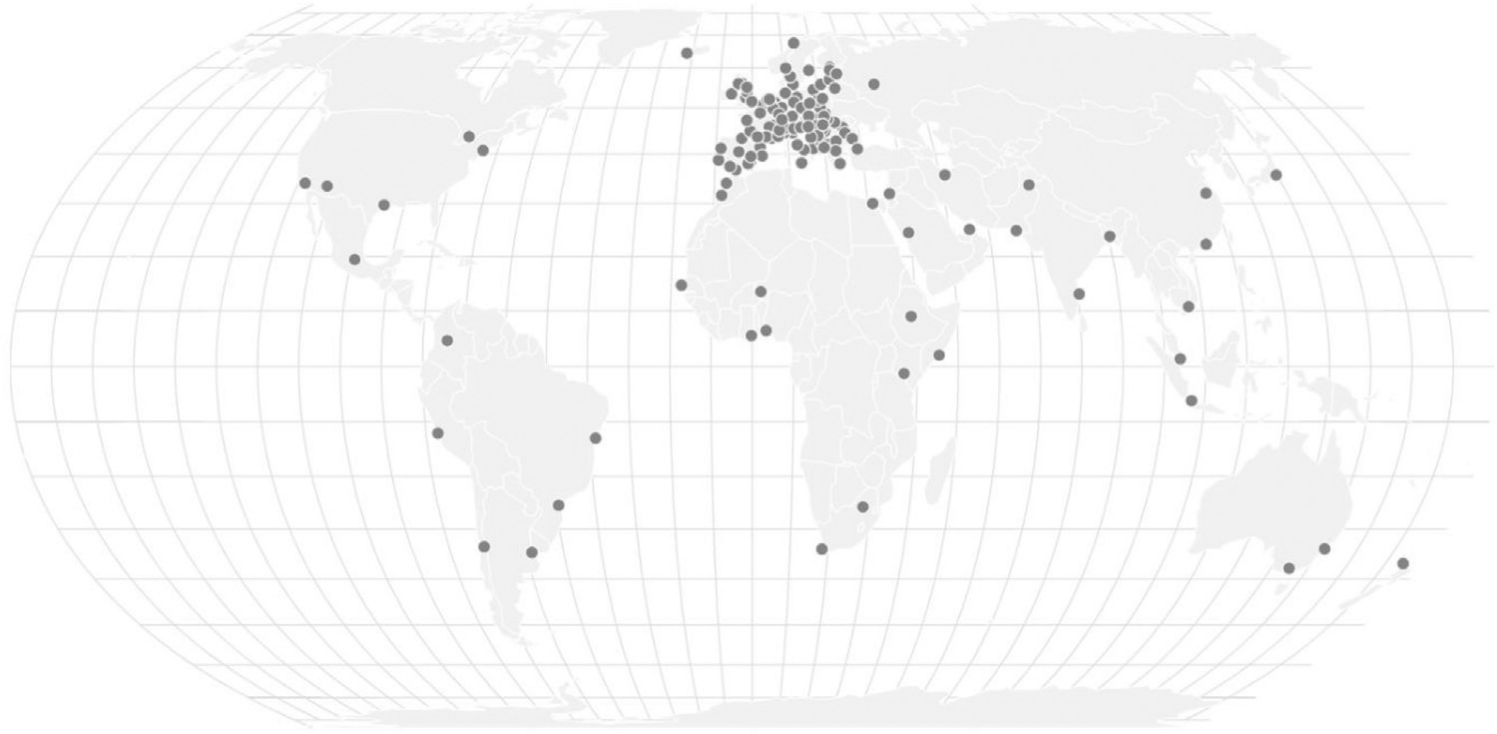
Overview map indicating the 142 cities for which present and future climate and heat stress data is made available.

**Fig. 2:**
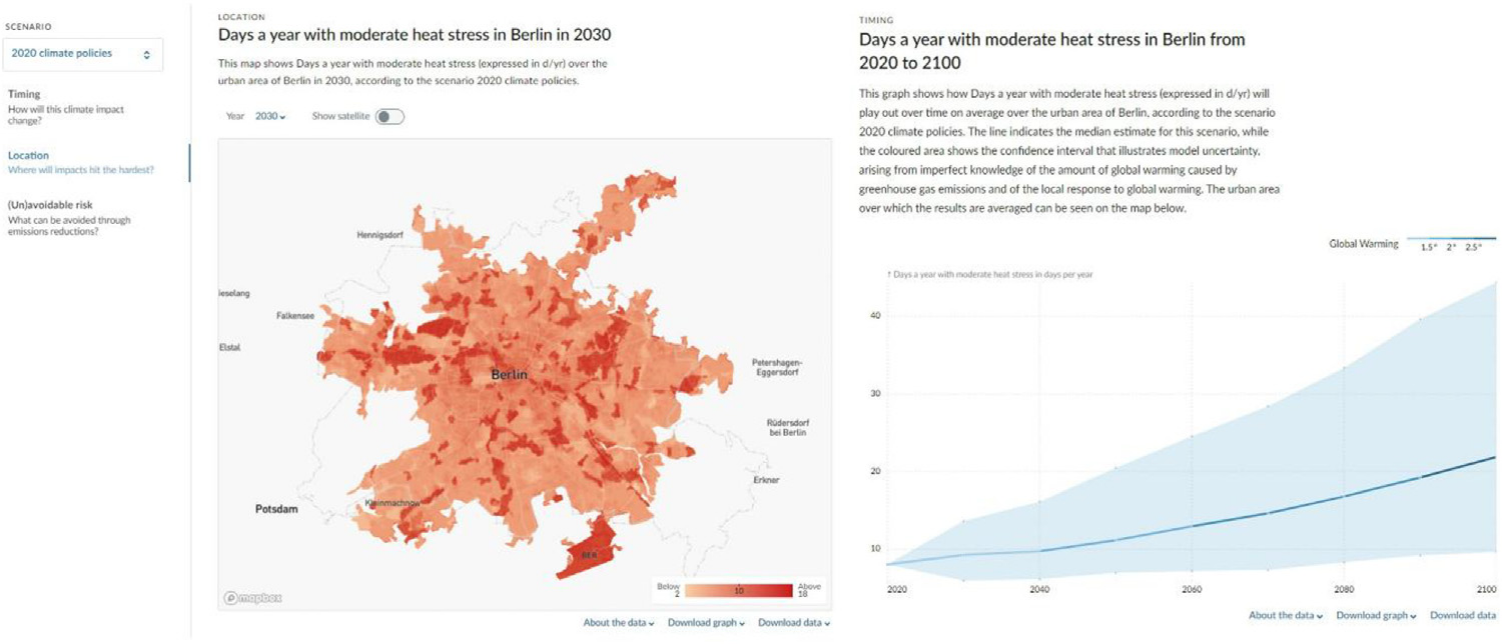
Snippet visualisations of the annual number of days with moderate heat stress (Wet Bulb Globe Temperatures above 25 °C) over Berlin in the 2020 climate policies scenario from a (left)) spatial and (right) temporal perspective. Visualisations obtained from the Climate Risk Dashboard.

Indicators for each decade are available for 142 cities spanning the period 2010–2100 for different future climate model scenarios and uncertainties (Fig. 1; Supplementary Table 1). The database of indicators is provided in both Geotiff and NetCDF format and is available in a local projection (which can be retrieved from the metadata of the files) and in EPSG:4326. Individual files and quick visualisations of indicator maps can be retrieved from the Climate Risk Dashboard (https://climate-risk-dashboard.iiasa.ac.at/impacts/explore), which allows a user to download the Geotiffs, NetCDFs and visualisations in PNG format (Fig. 2). A bulk data download option that allows downloading all indicators, time periods, scenarios at once is also provided via https://doi.org/10.5281/zenodo.13361538. As all data is georeferenced; users can visualise, analyse and manipulate the maps in GIS software tools and python.

An overview of the indicators calculated for each of the 142 cities is listed below.

- Temperature Indicators
  ○ Average daily maximum temperature: Average daily maximum 2 m temperature over the full decade
  ○ Average daily minimum temperature: Average daily minimum 2 m temperature over the full decade
  ○ Average daily temperature: Average daily 2 m temperature for the full decade
  ○ Maximum temperature of the warmest month [11]: The average maximum monthly temperature of the warmest month throughout the year
  ○ Maximum temperature of the coolest month [11]: The average minimum monthly temperature of the coolest month throughout the year
  ○ Daytime Urban Heat Island: The average difference in daily maximum temperatures between each pixel and rural temperature. The rural temperature is selected as the 10th percentile value of the non-urban pixels in the modelling domain (excluding water and wetland pixels). Temperatures are height-corrected by rescaling them to the average city height, applying a lapse rate of 6.5 K*km^−1^. It captures the difference in temperatures due to human activities and the modification of land surfaces.
  ○ Nighttime Urban Heat Island: The average difference in daily minimum temperatures between each pixel and rural temperature. The rural temperature is selected as the 10th percentile value of the non-urban pixels in the modelling domain (excluding water and wetland pixels). Temperatures are height-corrected by rescaling them to the average city height, applying a lapse rate of 6.5 K*km^−1^. It captures the difference in temperatures due to human activities and the modification of land surfaces.

- Temperature-based heat stress indicators
  ○ Annual heatwave days: A heatwave is defined as a minimum of three days in which both the daily maximum and minimum temperature exceed the 90th percentile threshold of a base period (taken as the period 2011–2020). The 90th percentile threshold is calculated over the full simulation domain (the city and its rural surroundings) based on the definition in Romanello et al. (2022) [12]. The indicator is depicted as the average number of heatwave days per year.
  ○ Annual heat-wave magnitude index daily (HWMId): The HWMId was defined by Russo et al. (2015) [13] and quantifies the magnitude of heatwaves by accounting for both their severity and duration, which makes it more suitable to compare extreme temperature events across the world as well as past, present and future heatwaves.
  ○ Annual number of days exceeding [25 °C; 30 °C; 35 °C]: Annual number of days in which the maximum temperature exceeds [25 °C; 30 °C; 35 °C].
  ○ Annual number of nights exceeding [20 °C; 25 °C; 28 °C]: Annual number of nights in which the minimum temperature does not drop below [20 °C; 25 °C; 28 °C]
  ○ Annual cooling degree hours: Cooling degree hours is an international standard to estimate energy usage for cooling dwellings using air conditioning. It is calculated as the number of hours during which the temperatures rises over 25 °C, multiplied by the number of degrees the temperature rises above 25 °C. The annual average value for the decade is shown.

- Wet Bulb Globe Temperature based heat stress indicators
  ○ Annual number of days WBGT > [25 °C; 28 °C; 29.5 °C; 31 °C]: Annual number of days in which the WBGT exceeds [25 °C; 28 °C; 29.5 °C; 31 °C] for at least one hour.
  ○ Annual number of nights WBGT > [25 °C; 28 °C]: Annual number of nights in which the WBGT does not drop below [25 °C; 28 °C]
  ○ Annual number of hours WBGT > [25 °C; 28 °C; 29.5 °C; 31 °C]: Annual number of hours in which the WBGT exceeds [25 °C; 28 °C; 29.5 °C; 31 °C].
  ○ Lost working hours (LWH) for intense activities: Depending on the WBGT, workers lose productivity or must take mandatory breaks. For intense activities (415 W; see ISO:7243 for examples) the following equation was constructed to calculate the lost productivity in one hour [14]:
    $$\mathrm{LWH}=1-\;\left\{ \begin{array}{cc} 1 & \mathrm{if}\;\mathrm{WBGT}\;<\;26.55918\\ -0.165*\mathrm{WBGT}+5.3982 & \mathrm{if}\;26.55918\;\leq \;\mathrm{WBGT}\;<\;32.59783\\ 0 & \mathrm{if}\;\mathrm{WBGT}\;\geq \;32.59783 \end{array}\right.$$
  ○ LWH for moderate activities: Depending on the WBGT, workers lose productivity or must take mandatory breaks. For moderate activities (300 W; see ISO:7243 for examples) the following equation was constructed to calculate the lost productivity in one hour [14]:
    $$\mathrm{LWH}=1-\;\left\{ \begin{array}{cc} 1 & \mathrm{if}\;\mathrm{WBGT}\;<\;28.2656\\ -0.2195*\mathrm{WBGT}+7.2043 & \mathrm{if}\;28.2656\;\leq \;\mathrm{WBGT}\;<\;32.82141\\ 0 & \mathrm{if}\;\mathrm{WBGT}\;\geq \;32.82141 \end{array}\right.$$
  ○ LWH for light activities: Depending on the WBGT, workers lose productivity or must take mandatory breaks. For light activities (180 W; see ISO:7243 for examples) the following equation was constructed to calculate the lost productivity in one hour [14]:
    $$\mathrm{LWH}=1-\;\left\{ \begin{array}{cc} 1 & \mathrm{if}\;\mathrm{WBGT}\;<\;31.0\\ -0.5*\mathrm{WBGT}+16.5 & \mathrm{if}\;31.0\;\leq \;\mathrm{WBGT}\;<\;33.0\\ 0 & \mathrm{if}\;\mathrm{WBGT}\;\geq \;33.0 \end{array}\right.$$

- Impact indicators
  ○ Population exposed to heatwave warning days
  ○ Population exposed to heat stress days

## 4 Experimental Design, Materials and Methods

The UrbClim model [15] is used to derive the hourly meteorological output to calculate the indicators. UrbClim is an urban boundary layer climate model, which is designed to dynamically downscale large-scale climate information to the extent of individual cities and their rural surroundings at very high resolution (up to 100 m). The UrbClim model consists of a land surface scheme containing simplified urban physics in which each model grid cell consists of a fraction of vegetation, bare soil and urban surface cover. This surface scheme is coupled to a 3-D atmospheric boundary layer module, taking into account the conservation equations of momentum, temperature, humidity and mass, while also specifically accounting for turbulent fluxes and the mixing layer. The atmospheric boundary layer is tied to synoptic-scale meteorological fields through the lateral and top boundary conditions, to ensure that the synoptic forcing is properly considered. A detailed description of the model physics can be retrieved from De Ridder et al. (2015) [15].

The historical simulation period spans a period of 10 years from 2008–2017. For this period, the UrbClim model is forced at its top and lateral boundaries by large-scale synoptic information from the ERA-5 reanalysis product (an overview of the variables that are used can be found in [15]. Apart from meteorological input data, the main strength of the UrbClim model lies in a detailed representation of the land surface properties. Depending on the region, different data sources have been used to characterise the urban surroundings (Supplementary Table 2). These data sources are resampled to the city modelling domains at 100 m spatial resolution to provide spatial heterogeneity within the urban canopy and define different surface parameters at the grid cell level, such as albedo, emissivity, building fraction, etc. Details on the approach can be found in [10,15].

The UrbClim model produces hourly output for meteorological variables such as temperature, humidity, wind speed, but also soil properties and energy fluxes at 100 m spatial resolution. Next to these basic meteorological variables, heat stress (Wet Bulb Globe Temperature) is calculated based on the model of Liljegren et al. (2008) [16]. This metric accounts for temperature, humidity and radiation and serves as a proxy for perceived temperature. It is calculated following ISO:7243 using the meteorological output of UrbClim and solar radiation information from the reanalysis dataset ERA-5. To accurately downscale radiation to 100 m resolution, a detailed representation of the building footprints and trees within the modelling domain is necessary. Their effect is two-fold. On the one hand, they cast shade, while on the other hand, buildings also absorb and emit radiation, adding an extra source of radiation. Detailed building footprint information is obtained from Open Street Map, Google Africa Buildings and Microsoft Building Footprints, while the fraction of trees in each 100 m pixel is defined depending on the land use.

The future climate forcing data is obtained from the MESMER-FaIR ensemble. Both FaIR and MESMER are climate model emulators that, with limited computational effort, can provide a large ensemble of climate model realisations. The FaIR emulator [17] is used to translate greenhouse gas emissions to the total strength of the forcing imposed on the climate system. This allows calculation of the (change in) Global Mean Temperature (GMT), constrained by both historic warming and expected future changes set out by the Intergovernmental Panel on Climate Change (IPCC). GMT is used by MESMER [18] to emulate the evolution of key climate variables over land for each of the given Earth temperature trajectories obtained from FaIR. In this work, the monthly downscaled module of MESMER is used, MESMER-M.

For each of the 142 cities, the following scenarios have been considered:

- 2020 climate policies (IPCC AR6 scenario): This scenario assumes that no further climate action is taken beyond the climate policies that were in place in 2020. Global warming reaches 2.9 °C in 2100 (best estimate), and would continue climbing into the new century.
- Delayed climate action (Gradual strengthening scenario in IPCC AR6): This scenario assumes that decarbonisation is delayed to the 2030s, but then takes place in earnest. Fossil fuel use never ends but is instead compensated for with high amounts of carbon dioxide removal. Global warming in 2100 reaches 1.7 °C (best estimate).
- Shifting pathway (IMP-SP scenario in IPCC AR6): This scenario explores how a broader shift towards sustainable development can be combined with stringent climate policies. Global warming peaks at 1.6 °C in 2060 and goes back to 1.3 °C in 2100 (best estimate).

The low spatial resolution and monthly temporal resolution prevents us from performing accurate future simulations of UrbClim dynamically driven by the MESMER-M ensemble. For example, changes in monthly average temperature might underestimate changes in the highest temperature quantiles (i.e. extreme temperatures generally change with higher amounts than average temperatures, which are of most interest in our study. To address this, we apply the quantile mapping bias algorithm [19]. Average monthly changes in temperature for each decade in the 2.5°x2.5° grid cell in which each city is located are obtained from MESMER-M, while changes in different temperature quantiles (10 in total) for different changes in monthly temperature are obtained from the CMIP6 archive, which has a higher time resolution (daily). It is used to calculate changes in quantiles of daily temperature for different levels of monthly temperature change within the city. These perturbations are added to the historical data simulated by UrbClim, leading to a time series of the same length and time scale as the historical time series but representative of future climate conditions. The approach above is applied for the three future climate scenarios forcing it with data from the mean, 5th and 95th percentiles of the ensemble of MESMER-M realisations until 2100. This provides an estimate of the uncertainty around the mean changes in the calculated indicators.

Apart from meteorological information that is directly obtained from the UrbClim model, impacts are computed using the open-sourced and open-access natural hazard risk model, CLIMADA (CLIMate ADAptation) [20]. The impact is calculated based on three components: hazard, exposure, and vulnerability. The hazard data is obtained from the city-scale meteorological modelling with the UrbClim model. The exposure is defined as the population and is obtained at 100 m spatial resolution from WorldPop for each of the 142 cities. The WorldPop Constrained Individual countries 2020 UN adjusted data provides the top-down constrained gridded population data which is adjusted to match the United Nations national estimate. Thus, the population data is validated for official reference. The original WorldPop dataset is on a country scale. To match the hazard data on a city scale, the original country-level gridded population data is trimmed into the specific city-level gridded population data. Vulnerability, such as age group, is not considered. Each person in the exposure has an equal weighting to the hazard.

## Limitations

An important limitation of the dataset is that it assumes static urban morphology. In reality, cities will expand and transform in ways that can influence both local climate responses and the number of people. By design, these factors are held constant to isolate the large-scale climate change signal at high resolution for present-day cities. The dataset should therefore be interpreted as a climatological baseline for assessing potential climate impacts, rather than as a projection of future urban conditions. Future extensions could combine this framework with urban growth scenarios and dynamic demographic projections to generate more application-oriented estimates of future urban climate risk.

**Fig. 3:**
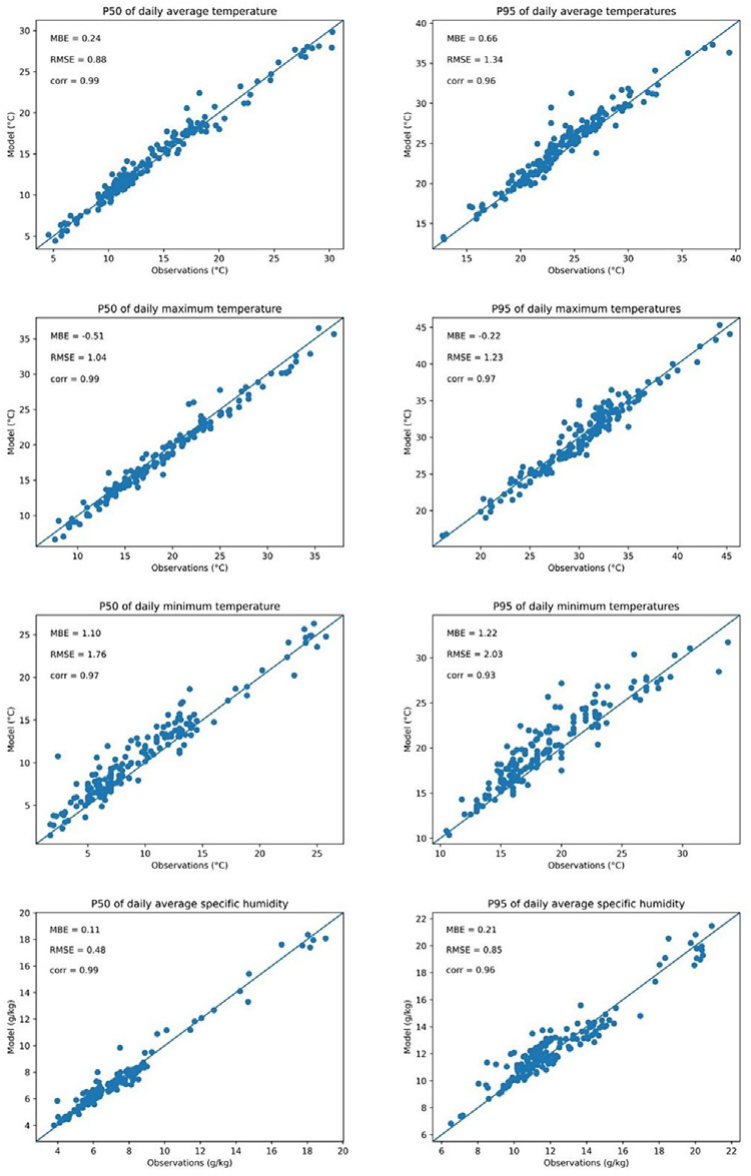
Scatter plots of modelled and observed daily average, maximum and minimum 2 m temperatures (top three rows) and daily average 2 m specific humidity (bottom row). 190 measurement stations representing 118 of the 142 cities were available. The 50th (left column) and 95th (right column) percentile are depicted, representing average and extreme conditions. Mean Bias Error (MBE), Root Mean Square Error (RMSE) and the Pearson correlation coefficient (corr) are shown for each panel.

The UrbClim validation is limited up to the extent of global meteorological observations from NOAA (NOAA National Centers of Environmental Information. 1999. Global Surface Summary of the Day - GSOD. 1.0.), which are located in rural environments and therefore provide limited validation of the urban heat island. Nevertheless, it provides a first-order idea of the representativeness of the model results. Both average and extreme (95th percentile) conditions were validated (Fig. 3). In general, daily maximum temperatures are represented accurately, with an average underestimation of 0.51 °C (for the 50th percentile) and 0.22 °C (for the 95th percentile) of the observed temperatures. With Root Mean Square Error (RMSE) values of respectively 1.04 and 1.23 and correlation coefficients of 0.99 and 0.97, the UrbClim model shows significant skill in representing daily maximum temperatures. Considering minimum temperatures, the UrbClim model is structurally warmer compared to observations with a Mean Bias Error (MBE) of 1.10 °C for the 50th percentile and 1.22 °C for the 95th percentile temperatures. This structural overestimation of minimum temperatures has been observed in previous validation studies of the UrbClim model and should be considered in the interpretation of the results. With respect to specific humidity, the UrbClim model achieves a very good agreement with the observational network, both for average and extreme conditions. With MBE of 0.11 *g*/kg and 0.21 *g*/kg and RMSE of 0.48 and 0.85 respectively, the model skilfully represents actual conditions. Despite only executed for rural locations, it puts confidence in the resulting indicators that are calculated by the model. It must be noted that the UrbClim model has been validated in urban contexts in different case studies (an overview can be found in https://urban-climate.eu/references).

Regarding future projections, the climate change signal is obtained from the MESMER-M ensemble. Although it provides a large sample of climate model projections, the monthly time frame, even though combined with daily CMIP6 values, might underestimate the future climate change impact of extremes, which should be accounted in the interpretation of future results. The uncertainty range provided for each of the future indicators is also solely based on the range provided by the MESMER-M ensemble. Although this provides a good estimate of future climate uncertainties, it should be noted that other sources of uncertainty are present (e.g. ERA-5).

Related to this, it must be noted that the results are highly dependent on the input datasets that are used, especially the ones defining the surface and the historical climate (ERA-5). Different input datasets (e.g. describing the surface in a slightly different way) might lead to different results.

## Ethics Statement

The authors have read and follow the ethical requirements for publication in Data Brief. We thereby confirm that the current work does not involve human subjects, animal experiments, or any data collected from social media platforms.

## CRediT Author Statement

**Niels Souverijns:** Conceptualization, Data Curation, Methodology, Formal Analysis, Writing. **Dirk Lauwaet:** Conceptualization, Methodology, Formal Analysis. **Quentin Lejeune:** Conceptualization, Methodology. **Chahan M. Kropf:** Data Curation, Formal Analysis. **Kam Lam Yeung:** Data Curation, Formal Analysis. **Shruti Nath:** Data Curation, Formal Analysis. **Carl F. Schleussner:** Conceptualization, Funding acquisition. All authors reviewed the manuscript.

## Acknowledgments

This project has received funding from the European Union’s Horizon Europe research and innovation programme under grant agreement No 101,003,687.

## Declaration of Competing Interest

The authors declare that they have no known competing financial interests or personal relationships that could have appeared to influence the work reported in this paper.

## Data Availability

Zenodo100m climate and heat stress information up to 2100 for 142 cities around the globe (Original data)

## Appendix Supplementary materials

Image, application 1

Image, application 2
